# The Conceptualization and Measurement of Research Impact in Primary Health Care: Protocol for a Rapid Scoping Review

**DOI:** 10.2196/55860

**Published:** 2024-04-23

**Authors:** Monica Aggarwal, Brian G Hutchison, Kristina M Kokorelias, Vivian R Ramsden, Noah M Ivers, Andrew Pinto, Ross E G Uphsur, Sabrina T Wong, Nick Pimlott, Steve Slade

**Affiliations:** 1 Dalla Lana School of Public Health University of Toronto Toronto, ON Canada; 2 Department of Family Medicine McMaster University Hamilton, ON Canada; 3 Department of Health Research Methods, Evidence, and Impact McMaster University Hamilton, ON Canada; 4 Rehabilitation Sciences Institute University of Toronto Toronto, ON Canada; 5 Section of Geriatrics Sinai Health Toronto, ON Canada; 6 Department of Academic Family Medicine University of Saskatchewan Saskatoon, SK Canada; 7 Department of Family and Community Medicine Faculty of Medicine University of Toronto Toronto, ON Canada; 8 Women’s College Hospital Toronto, ON Canada; 9 Upstream Lab, MAP Centre for Urban Health Solutions, Li Ka Shing Knowledge Institute Unity Health Toronto Toronto, ON Canada; 10 Department of Family and Community Medicine St. Michael’s Hospital Toronto, ON Canada; 11 School of Nursing and Centre for Health Services and Policy Research University of British Columbia Vancouver, BC Canada; 12 Research Department College of Family Physicians of Canada Mississuaga, ON Canada

**Keywords:** research impact, primary health care, measurement, definition, concept, development, implementation, health policy, policy, health service, rapid review, review, research, policies, societal, productivity, literature database

## Abstract

**Background:**

The generation of research evidence and knowledge in primary health care (PHC) is crucial for informing the development and implementation of interventions and innovations and driving health policy, health service improvements, and potential societal changes. PHC research has broad effects on patients, practices, services, population health, community, and policy formulation. The in-depth exploration of the definition and measures of research impact within PHC is essential for broadening our understanding of research impact in the discipline and how it compares to other health services research.

**Objective:**

The objectives of the study are (1) to understand the conceptualizations and measures of research impact within the realm of PHC and (2) to identify methodological frameworks for evaluation and research impact and the benefits and challenges of using these approaches. The forthcoming review seeks to guide future research endeavors and enhance methodologies used in assessing research impact within PHC.

**Methods:**

The protocol outlines the rapid review and environmental scan approach that will be used to explore research impact in PHC and will be guided by established frameworks such as the Canadian Academy of Health Sciences Impact Framework and the Canadian Health Services and Policy Research Alliance. The rapid review follows scoping review guidelines (PRISMA-ScR; Preferred Reporting Items for Systematic Review and Meta-Analysis Extension for Scoping Reviews). The environmental scan will be done by consulting with professional organizations, academic institutions, information science, and PHC experts. The search strategy will involve multiple databases, citation and forward citation searching, and manual searches of gray literature databases, think tank websites, and relevant catalogs. We will include gray and scientific literature focusing explicitly on research impact in PHC from high-income countries using the World Bank classification. Publications published in English from 1978 will be considered. The collected papers will undergo a 2-stage independent review process based on predetermined inclusion criteria. The research team will extract data from selected studies based on the research questions and the CRISP (Consensus Reporting Items for Studies in Primary Care) protocol statement. The team will discuss the extracted data, enabling the identification and categorization of key themes regarding research impact conceptualization and measurement in PHC. The narrative synthesis will evolve iteratively based on the identified literature.

**Results:**

The results of this study are expected at the end of 2024.

**Conclusions:**

The forthcoming review will explore the conceptualization and measurement of research impact in PHC. The synthesis will offer crucial insights that will guide subsequent research, emphasizing the need for a standardized approach that incorporates diverse perspectives to comprehensively gauge the true impact of PHC research. Furthermore, trends and gaps in current methodologies will set the stage for future studies aimed at enhancing our understanding and measurement of research impact in PHC.

**International Registered Report Identifier (IRRID):**

PRR1-10.2196/55860

## Introduction

High-performing primary health care (PHC) is recognized as the cornerstone of robust health care systems [1,2]. High-income countries often possess robust health care systems with well-established PHC organizations and research institutions, making them pivotal in shaping global health policies and practices [3,4]. High-quality research identifying what is needed to strengthen the performance of PHC organizations and their integration with each other and the broader health system is essential to inform the sustainable development of health care [5]. A PHC orientation to health service research strives to understand the influence of health’s socioeconomic, physical, biological, and cultural determinants within the relevant broader political, sociohistorical, and economic contexts. PHC services can improve health and health services delivery, which could result in improved individual, community, and population health outcomes.

The need for PHC research to determine the efficacy of treatment and test theories and develop new models of care has been documented [6]. PHC encompasses primary care, disease prevention, health promotion, population health, and community development within a holistic framework to provide essential community-focused health care [7,8]. We operationalize “PHC research” to refer to studies that investigate a broad range of topics related to preventive care, health promotion, diagnosis, treatment, ongoing management of common illnesses and chronic conditions, and the social determinants of health within the context of PHC settings [9].

There are several conceptual frameworks and approaches that have been developed for research impact assessment [10]. In Canada, the Canadian Academy of Health Sciences (CAHS) Impact Framework has been adapted to examine the impact of investments in health research. The CAHS Impact Framework uses 5 impact categories: advancing knowledge, capacity building, informing decision-making, health impacts, and socioeconomic impacts and provides a menu of nearly 70 indicators that map onto these domains [11]. In 2018, the Canadian Health Services and Policy Research Alliance (CHSaPRA) was developed by the Canadian health research community based on CAHS to guide the assessment of the impact of research on decision-making [12].

Despite the development of these frameworks, little is known about how research impact is conceptualized in PHC [13]. Globally, PHC research is a small proportion of research output [14]. While existing reviews address research impact within the health care and health service research context [15,16], these reviews are not tailored to consider the unique functions of PHC. As noted by the Council of Academic Family Medicine, PHC research is unique since it involves the delivery of care to patients across the care life cycle, which includes disease prevention, health promotion, and chronic care management [17-20]. It also provides evidence that is unique for the organization and delivery of care, evaluation of innovations, translation of research into practice, and participatory action and community-based approaches [17]. Due to the broader effects of research on patients, practices, services, population health, community, and policy formulation, a dedicated review in PHC is essential to broaden our understanding of research impact, including nuances of how it may or may not be different than other health services research. To establish directions to evolve definitions of research productivity in the context of PHC, funders, academic institutions, researchers, and the public need a better understanding of “research impact” in PHC.

Several scoping reviews, such as those conducted by Murphy et al [21] on measurement in rural PHC, Noorihekmat et al [22] on performance measurement frameworks in public health and primary care systems, and Akl et al [23] on faculty productivity in academic medical centers, have explored aspects of research productivity and measurement within health care. However, to the best of our knowledge, there is no existing review that has offered a focused exploration of the specific conceptualizations and measures of research impact within the context of PHC. Such a review would offer a unique and detailed analysis that aims to elucidate the nuances and intricacies of research productivity, thereby contributing a distinct perspective to the existing literature.

This review protocol aims to fill a gap in knowledge by examining the scientific literature on research impact in the context of PHC. Drawing from established frameworks like the CAHS Impact Framework [24] and CHSaPRA [25], we will aim to (1) elucidate the various conceptualizations of research impact within the context of PHC and (2) identify measures of research impact used in the PHC literature by PHC researchers. The study will contribute to understanding and identifying trends in how the impact is understood and measured, highlighting existing gaps or areas needing further investigation within this domain. The findings of the study will be leveraged to inform a future study that will explore the perspectives of patients, citizens, community groups representing equity-deserving groups, PHC leaders, researchers, and policy makers on the definition and measurement of research impact in PHC.

## Methods

### Study Design

A rapid scoping review [26] and environmental scan [27] will be conducted. A rapid scoping review methodology is suitable for this investigation, as it allows for a comprehensive but expedited exploration of the existing literature surrounding research impact within PHC. Given the breadth of the topic and the need to capture a wide array of literature quickly, the rapid scoping review methodology aligns with the urgency to understand conceptualizations and measurements of research impact in PHC [26]. This approach permits the incorporation of diverse study designs, including gray literature and various publication types, facilitating a thorough investigation of research impact concepts within a condensed timeframe [26]. However, the rapid review will be informed by existing guidelines for scoping reviews [28] and the Arksey and O’Malley [29] methodological steps (stages 1 to 5 described below), aiming for standardized execution and reporting and enhancing the credibility of the findings. Scoping reviews aim to map fundamental concepts in a research area, define key terms, and delineate conceptual limits, making it suitable for our purposes [29,30]. The CRISP (Consensus Reporting Items for Studies in Primary Care) statement proposed by Sturgiss et al [31] will be used for the comprehensive extraction and reporting in PHC research to enable the analysis of practices and policies across a diverse range of countries and territories.

Similar to a rapid review, an environmental scan is often used by institutions to collect information [27]. An environmental scan is a systematic approach to gathering and analyzing information from various sources beyond traditional academic literature, including publicly available data, unpublished reports, and consultations with experts [32]. It aims to comprehensively identify relevant information, trends, and developments within a specific field or context [32]. Researchers can also use an environmental scan to identify current and potential research needs and trends to enhance decision-making [27]. While there are various methodologies and sources for collecting and analyzing information for an environmental scan [32], our environmental scan will be conducted by identifying professional organizations, academic institutions, and experts in the fields of information science and PHC to begin our search.

We will adhere the reporting of our review to the PRISMA-ScR (Preferred Reporting Items for Systematic Review and Meta-Analysis Extension for Scoping Reviews) reporting guidelines [33], as there are currently no existing guidelines for rapid reviews. The study will be conducted in 2024 with results anticipated by the end of the year. The research team is comprised of PHC researchers (clinicians, PhD trained) or leaders in the field.

### Stage 1: Identifying the Research Questions

We intend to address the following research questions: (1) How is research impact conceptualized in PHC? (2) How is research impact measured in PHC? (3) What methodological or conceptual frameworks are used to evaluate PHC research impact? What are the benefits and challenges of assessing PHC research impact?

### Stage 2: Identifying Relevant Studies

We will work with an information specialist to develop a search strategy for the following academic databases: PsycINFO, MEDLINE, Embase, and CINAHL Plus. These databases were intentionally selected for their inclusion of PHC literature and thus are likely to capture relevant scholarly material. The strategy will initially be applied in MEDLINE before being adapted for other databases. We will also search for literature in Google Scholar to “the wide range of resources including papers from academic journals, conference papers, theses, and dissertations” [34]. The peer-reviewed search strategy will also be used in Google Scholar. A citation search of the reference lists of selected papers will be conducted to ensure that a wider scope of papers is included. Forward citation searching will also be done for literature that cites eligible studies included in the review [35]. Scopus, Web of Science, and Google Scholar will be used for forward citation tracking to ensure a comprehensive search.

To ensure a comprehensive review of relevant sources and databases, relevant gray literature databases, catalogs, and search engines (eg, Google, OpenGrey, and TripPro) will be hand searched across high-income countries. White papers will also be explored through health care–focused think-tank websites. We will also contact librarians in the field of PHC and information specialists through several mailing lists (including Canadian Medical Libraries and expert searching through the College of Family Physicians of Canada) to ask for further studies or gray literature. We define gray literature as “that which is produced on all levels of government, academics, business and industry in print and electronic formats, but which is not controlled by commercial publishers” [36].

An environmental scan of institutes and initiatives that follow their strategic funding to understand research impacts, such as the Canadian Institute for Health Research’s Community-Based Primary Health Care reports and the SPOR Evidence Alliance [37] will also be conducted. We also aim to examine metrics used by funding agencies within jurisdictions. We will contact each one individually for any published or unpublished evaluations of these PHC activities and ask for any other organizations or experts, who may help us to find as many materials as possible.

The search strategies for the databases were devised in collaboration with the 2 information specialists (Multimedia Appendix 1). Before the literature search and screening process, reviewers will receive training from the principal investigator (MA) to ensure a foundational grasp of the field’s background and the review’s objectives.

### Stage 3: Study Selection

Two research assistants (one of whom is KMK) will perform all searches in the databases, citation searching, as well as the environmental scan. Findings from all databases will be amalgamated and imported into Covidence (Veritas Health Innovation) for streamlined documentation and management of studies throughout the review process [38]. Additionally, any duplicate publications will be eliminated.

For the gray literature and environmental scan, the research assistants will independently conduct searches across the sources. The research assistants will document the sources and databases accessed for gray literature, specifying the search terms, strategies, and any limitations applied. Any date range, filters, and criteria used to identify relevant gray literature sources will be noted. The research assistants will document the results obtained, including the number of documents retrieved, and provide a clear account of any exclusions made along with justifications. During frequent team meetings, the research team will discuss how duplicates were managed. This documentation should ensure transparency and reproducibility, allowing others to follow and validate the search methodology [39,40]. Gray literature will be organized and managed in a structured Microsoft Excel (Microsoft Corp) spreadsheet. Microsoft Excel will also be used to organize details of the environmental scan, such as the institute or initiative names, contact information, links to relevant reports or evaluations, dates of contact, and any additional notes or follow-up actions required.

We will include gray and scientific literature of any study design that (1) have an explicit focus on the research impact, (2) explicitly focus on PHC research, and (3) are published from a high-income country (defined as per the World Bank classification [41], ie, Andorra, Antigua and Barbuda, Aruba, Australia, Austria, Bahamas, Bahrain, Barbados, Belgium, Bermuda, British Virgin Islands, Brunei Darussalam, Canada, Cayman Islands, Channel Islands, Chile, Croatia, Curaçao, Cyprus, Czech Republic, Denmark, Estonia, Faroe Islands, Finland, France, French Polynesia, Germany, Gibraltar, Greece, Greenland, Guam, Hong Kong SAR China, Hungary, Iceland, Ireland, Isle of Man, Israel, Italy, Japan, Republic of Korea, Kuwait, Latvia, Liechtenstein, Lithuania, Luxembourg, Macao SAR China, Malta, Monaco, Nauru, Netherlands, New Caledonia, New Zealand, Northern Mariana Islands, Norway, Oman, Palau, Poland, Portugal, Puerto Rico, Qatar, San Marino, Saudi Arabia, Seychelles, Singapore, Sint Maarten (Dutch part), Slovakia, Slovenia, Spain, St Kitts and Nevis, St Martin (French part), Sweden, Switzerland, Taiwan China, Trinidad and Tobago, Turks and Caicos Islands, the United Kingdom, United Arab Emirates, Uruguay, the United States, and Virgin Islands (United States).

We define an “explicit focus on the research impact” as studies or papers where the primary or significant emphasis is placed on evaluating, measuring, or discussing the effects, outcomes, influence, or implications of research activities or interventions in the field of PHC or by PHC providers. This includes studies using both formal methodological frameworks, such as the CAHS Impact Framework, and ad hoc approaches using single or limited metrics. This could involve investigations into the tangible outcomes or effects of research endeavors such as changes in health care practices, policy implications, patient outcomes, health system improvements, or societal impacts resulting from PHC research initiatives [42]. Additionally, studies proposing frameworks for evaluating PHC research impact, regardless of whether they are empirically trialed or piloted, are considered, recognizing the value of theoretical advancements in this domain. However, studies predominantly focused on assessing the impact of health care interventions themselves, rather than the research process or outcomes, are excluded to ensure a manageable scope and relevance to the review objectives.

Limiting inclusion to studies published from high-income countries aligns with the rapid nature of this review while ensuring a focus on PHC research that reflects contexts, systems, and health care settings that share similar socioeconomic and health care infrastructure characteristics.

Only literature from 1978 (signing of the Alma-Ata Declaration [43]) to the present day and published in English will be considered. Research conducted within this timeframe ensures relevance to contemporary PHC practices and current understanding but also allows for the inclusion of recent advancements, methodologies, and perspectives in the field. Setting a specific timeframe and language criteria can help manage the volume of literature to be reviewed, which is key in a rapid review [26,44].

The results from all databases will be imported into EndNote (Clarivate Analytics), and all duplicates will be removed [45]. The unique papers will then be added to Covidence software to help facilitate screening, study selection, and extraction [38]. After generating a list of papers from our search strategy, we will engage in a 2-stage screening process with at least 1 independent reviewer at each stage. In the first stage, 2 trained, independent reviewers will screen papers for suitability based on their titles and abstracts in duplicate. We will report on the calculation of interrater reliability using Cohen κ coefficient [46] to assess the consistency of screening decisions between reviewers and ensure the reliability of the study selection process. A third reviewer will review 25% of the excluded papers to ensure no papers were inadvertently excluded. If there is ambiguity on whether specific papers fit the scope of this protocol, the principal investigator (MA) will be consulted. In the second stage, single reviewer (KMK) will conduct full-text reviews of the potentially eligible studies using the inclusion criteria aforementioned [47]. Again, a third reviewer will review 25% of the excluded papers to ensure no papers were inadvertently excluded. Throughout the process, disagreements between reviewers regarding the inclusion or exclusion of papers will be resolved via discussion with the principal investigator (MA), who will advise the reviewers of the outcome (ie, include or exclude). Given the iterative nature of scoping reviews, the inclusion and exclusion criteria will be refined (eg, added specificity), if needed, after increased familiarity with the data.

The comprehensive outcomes of both the searches (ie, databases and gray literature, including the environmental scan) and the study inclusion procedure will be thoroughly detailed in the final report of the review. These will be articulated using a PRISMA-ScR flow diagram, ensuring a clear and structured presentation of the search process and the selection of studies for the review [33].

### Stage 4: Charting the Data

The research team will develop initial charting variables based on the research questions and the CRISP statement [48]. The preliminary variables that will be extracted from the studies will include (1) authors, (2) year, (3) country where the study was conducted or country of first author’s affiliation, (4) journal, (5) methodology of paper (including whether a framework or ad hoc approach is used for measuring impact), (6) definition or conceptualization of impact (including how the impact is measured), (7) notice of research team’s primary care experience and collaboration, (8) description of the study participants and populations in the context of primary care, (9) description of the primary care team, (10) description of the conditions under study in the context of primary care outcome measures (primary and secondary), (11) units of analysis, (12) findings, and (13) recommendations or discussion (eg, gaps, challenges or barriers, recommendations, and evidence-based or best practices).

Two team members will independently chart the first 5 papers that meet our inclusion criteria and refine the definitions for the variables or charting categories if necessary. The research team will discuss the extracted data. If consensus is reached, 1 researcher (KMK) will extract data from the remaining papers. If consensus is not reached, the 2 individuals will continue to extract 1 paper in duplicate until consensus is reached. All discrepancies between reviewers will be addressed through discussion and by involving additional individuals. The charted data will be organized and presented in a Microsoft Excel spreadsheet.

### Stage 5: Collating, Summarizing, and Reporting the Results

To achieve our aims, we will adopt 3 distinct strategies for reporting and presentation. Initially, the research team will use a PRISMA-ScR checklist to ensure systematic reporting of our methods and screening processes [33]. Additionally, the charted data will be reviewed, synthesized, and analyzed through a numerical summary analysis that will include an overview of study characteristics and help to identify predominant conceptualizations and measurement frameworks used for research impact in PHC [29]. A directed content analysis will be carried out on the extracted data [49]. This method entails identifying specific concepts, definitions, methodological approaches, recommendations, benefits, and challenges associated with research impact in PHC. A coding framework will be developed based on established theories and frameworks relevant to PHC research impact, ensuring that the analysis remains focused and aligned with the study objectives. Each piece of extracted data will be systematically coded according to predefined categories, allowing for consistent and structured analysis. Through the directed content analysis, key themes extracted from the selected papers will be categorized, summarized, and presented using a narrative synthesis [47] that describes how research impact is conceptualized and measured in PHC. This analysis will also encompass definitions of research impacts, methodological techniques to measure research impact, and recommendations for improving PHC research impact and identify benefits or challenges of measurements of research impact in the context of PHC.

Finally, the synthesis will address strengths, study limitations, existing knowledge gaps, and potential avenues for future research pertinent to research impact in the realm of PHC. This is aligned with the goals of scoping reviews, which aims to comprehensively outline the scope and characteristics of existing literature [29]. However, as consistent with the scoping review methodology, we anticipate that the narrative synthesis will be an iterative process and dependent on the literature found [47].

## Results

The results of this study are expected in December 2024. The dissemination of findings from this rapid scoping review on the conceptualization and measurement of research impact in PHC will ensure that the insights generated are shared with relevant stakeholders and contribute to informed decision-making and further research efforts.

The findings of this scoping review will be disseminated through various channels to reach a wide audience. A paper detailing the methodology, findings, and implications of the rapid review and environmental scan will be submitted to a relevant peer-reviewed journal in the field of PHC, family medicine, health services research, or impact assessment (eg, *Journal of Primary Care & Community Health*, *BMC Family Practice*, *Health Services Research*, *Journal of Health Services Research & Policy*, and *The Annals of Family Medicine*). A concise and accessible policy brief summarizing the key findings and their implications for policy makers, stakeholders, and PHC leaders will be developed and distributed through appropriate channels (eg, College of Family Physicians of Canada, Canadian Institute for Health Information, Canadian Foundation for Healthcare Improvement, Canadian Institutes of Health Research, Canadian Primary Care Sentinel Surveillance Network, and Canadian Primary Care Research Network). The research team will present the findings at relevant academic conferences, seminars, and workshops attended by researchers, practitioners, and policy makers in the fields of PHC and health services research (eg, North American Primary Care Research Group, Canadian Association of Health Services and Policy Research, Society for Academic Primary Care, and International Conference on Primary Health Care). Collaboration with existing networks and initiatives in PHC research and impact assessment will be sought to integrate the findings into ongoing discussions and efforts. The dissemination materials will be tailored to the needs and interests of different stakeholders. For example, academic publications will provide detailed methodology and findings for researchers, while policy briefs will focus on practical implications for decision makers. We will also create 1-page infographics for patient and public communities (eg, National Association of Community Health Centers). Presentations and webinars will be customized to engage different audiences effectively.

## Discussion

### Preliminary Findings

The preliminary findings from this study have yet to be compiled and analyzed as the review is ongoing. The forthcoming rapid review and environmental scan on research impact in PHC will help to elucidate conceptualizations and measurement methodologies of impact, shaping the understanding of research impact within this domain. By rigorously and systematically reviewing a breadth of literature sources, this study aspires to unravel the diverse perspectives and approaches used to gauge the impact of research activities in PHC. This review will be able to discern trends, illuminate potential gaps, and outline areas necessitating further exploration or refinement in the assessment of research impact. Such delineations are envisioned to inform future research, policy considerations, and practice innovations in medicine and academia, ultimately contributing to the continuous enhancement of PHC services and policies in PHC settings.

### Limitations

The methodology and methods outlined for the scoping review and environmental scan present several potential limitations. First, the selection process might carry biases due to restrictions such as language and publication date, possibly excluding valuable insights from diverse settings or languages. We acknowledge that relying solely on the World Bank’s classification system for high-income countries may oversimplify the diversity of socioeconomic and health care infrastructure characteristics among nations and may not consider the varied health care contexts, systems, and priorities among high-income countries that could in turn influence research impact.

Additionally, researcher biases might influence study interpretation or selection, potentially affecting the review’s credibility. Second, while the rapid review approach aids efficiency, it may compromise a comprehensive understanding of nuanced concepts related to research impact in PHC. Third, accessing relevant unpublished materials in gray literature might pose challenges, potentially affecting the credibility or reliability of findings.

### Conclusions

This protocol outlines a rapid scoping review and environmental scan for exploring research impact in PHC, which will provide critical insights for advancing PHC systems. Recognizing PHC’s pivotal role in health care, this study underscores the need for comprehensive research to bolster PHC organizations’ performance and integration within broader health systems. Drawing from established frameworks such as CAHS and CHSaPRA, the forthcoming review will examine how research impact is conceptualized and measured in PHC, addressing an existing gap in knowledge. Emphasizing the scarcity of recent reviews specifically focused on this domain, this upcoming study pioneers a comprehensive analysis, shedding light on the nuances of research impact. By delineating varied conceptualizations of research impact and measures used in PHC, this review charts a pathway for future research, potentially refining measurement methodologies and informing decision-making for stakeholders in the PHC landscape.

While findings are pending, the study aspires to inform future research endeavors, policy formulations, and practice enhancements in the realm of PHC, which has a pivotal role within health care systems both provincially and federally, for example, Indigenous people living on reserve and people living in prisons.

## Appendix

Multimedia Appendix 1 Search strategy.

## Abbreviations

CAHS: Canadian Academy of Health Sciences
CHSaPRA: Canadian Health Services and Policy Research Alliance
CRISP: Consensus Reporting Items for Studies in Primary Care
PHC: primary health care
PRISMA-ScR: Preferred Reporting Items for Systematic Review and Meta-Analysis Extension for Scoping Reviews

## Data Availability

The data sets generated and analyzed during this study will be made available from the corresponding author on reasonable request.
