# Supplementary material for: The Conceptualization and Measurement of Research Impact in Primary Health Care: Protocol for a Rapid Scoping Review
Source: JMIR Res Protoc. 2024 Apr 23;13:e55860. doi: 10.2196/55860 (PMC11077413; doi:10.2196/55860)
Supplement: Multimedia Appendix 1 [file resprot_v13i1e55860_app1.docx]

**Multimedia Appendix 1. Search strategy.**

**MEDLINE**

Ovid MEDLINE: Epub Ahead of Print, In-Process & Other Non-Indexed Citations, Ovid MEDLINE® Daily and Ovid MEDLINE® <1946-Present>

1 *primary health care/ or *patient-centered care/ 68589
2 *Patient Care Team/ 29268
3 *preventive health services/ or *primary prevention/ 18481
4 *general practice/ or *family practice/ 52256
5 (Primary Healthcare* or Primary Health Care* or Primary Care* or Patient Centered Care* or Person Centered Care* or Patient Focused Care* or Patient Centered Nursing* or Patient Care Team* or Multidisciplinary Care Team* or Multidisciplinary Health Team* or Medical Care Team* or Healthcare Team* or Health Care Team* or Interdisciplinary Health Team* or Inter-disciplinary Health Team* or Interdisciplinary Care Team* or Inter-disciplinary care Team* or Preventive Health Care* or Preventive Healthcare* or Preventive Health* or Preventive Health Service* or Family Practice* or General Practice* or Comprehensive Healthcare* or Comprehensive Health Care*).ab,ti. 241832
6 1 or 2 or 3 or 4 or 5 329557
7 ((research adj3 impact*) or (research adj3 signif*) or (research adj3 import*) or (research adj3 influen*) or (research adj3 reperc*) or (research adj3 conseq*) or (research adj3 effect*) or (research adj3 outcome*) or (research adj3 implic*) or (research adj3 ramif*) or h-index* or bibliometric* or altmetric* or Science of Implementation* or bibliography of medicine).ab,ti. 134769
8 *health services research/ 16026
9 *Translational Research, Biomedical/ 6984
10 *interdisciplinary research/ or *public health systems research/ or *research report/ 3182
11 7 or 8 or 9 or 10 159061
12 6 and 11 4697
13 limit 12 to (english language and yr="1970 -Current") 4494

**PSYCHINFO**

1 *primary health care/ or *patient-centered care/ 68589
2 *prevention/ or *preventive health services/ 9138
3 *Family Medicine/ 0
4 (Primary Healthcare* or Primary Health Care* or Primary Care* or Patient Centered Care* or Person Centered Care* or Patient Focused Care* or Patient Centered Nursing* or Patient Care Team* or Multidisciplinary Care Team* or Multidisciplinary Health Team* or Medical Care Team* or Healthcare Team* or Health Care Team* or Interdisciplinary Health Team* or Inter-disciplinary Health Team* or Interdisciplinary Care Team* or Inter-disciplinary care Team* or Preventive Health Care* or Preventive Healthcare* or Preventive Health* or Preventive Health Service* or Family Practice* or General Practice* or Comprehensive Healthcare* or Comprehensive Health Care*).ab,ti. 241832
5 1 or 2 or 3 or 4 270733
6 ((research adj3 impact*) or (research adj3 signif*) or (research adj3 import*) or (research adj3 influen*) or (research adj3 reperc*) or (research adj3 conseq*) or (research adj3 effect*) or (research adj3 outcome*) or (research adj3 implic*) or (research adj3 ramif*) or h-index* or bibliometric* or altmetric* or Science of Implementation* or bibliography of medicine).ab,ti. 134769
7 *public health research/ 0
8 *interdisciplinary research/ or *public health systems research/ or *research report/ 3182
9 6 or 7 or 8 137842
10 5 and 9 2960
11 limit 10 to (english language and yr="1970 -Current") 2887

**CINAHL**

1 (MM "Primary Health Care")

2 (MM "Patient Centered Care")

3 (MM "Preventive Health Care")

4 (MM "Family Practice")

5 (MM "Multidisciplinary Care Team")

6 (TI “Primary Healthcare*” or “Primary Health Care*” or “Primary Care*” or “Patient Centered Care*” or “Person Centered Care*” or “Patient Focused Care*” or “Patient Centered Nursing*” or “Patient Care Team*” or “Multidisciplinary Care Team*” or “Multidisciplinary Health Team*” or “Medical Care Team*” or “Healthcare Team*” or “Health Care Team*” or “Interdisciplinary Health Team*” or “Inter-disciplinary Health Team*” or “Interdisciplinary Care Team*” or “Inter-disciplinary care Team*” or “Preventive Health Care*” or “Preventive Healthcare*” or ‘Preventive Health*” or “Preventive Health Service*” or “Family Practice*” or “General Practice*” or “Comprehensive Healthcare*” or “Comprehensive Health Care*”)

7 (AB “Primary Healthcare*” or “Primary Health Care*” or “Primary Care*” or “Patient Centered Care*” or “Person Centered Care*” or “Patient Focused Care*” or “Patient Centered Nursing*” or “Patient Care Team*” or “Multidisciplinary Care Team*” or “Multidisciplinary Health Team*” or “Medical Care Team*” or “Healthcare Team*” or “Health Care Team*” or “Interdisciplinary Health Team*” or “Inter-disciplinary Health Team*” or “Interdisciplinary Care Team*” or “Inter-disciplinary care Team*” or “Preventive Health Care*” or “Preventive Healthcare*” or ‘Preventive Health*” or “Preventive Health Service*” or “Family Practice*” or “General Practice*” or “Comprehensive Healthcare*” or “Comprehensive Health Care*”)

8 1 or 2 or 3 or 4 or 5 or 6 or 7

9 (TI (research N3 impact*) or (research N3 signif*) or (research N3 import*) or (research N3 influen*) or (research N3 reperc*) or (research N3 conseq*) or (research N3 effect*) or (research N3 outcome*) or (research N3 implic*) or (research N3 ramif*) or h-index* or bibliometric* or altmetric* or Science of Implementation* or bibliography of medicine)

10 (AB (research N3 impact*) or (research N3 signif*) or (research N3 import*) or (research N3 influen*) or (research N3 reperc*) or (research N3 conseq*) or (research N3 effect*) or (research N3 outcome*) or (research N3 implic*) or (research N3 ramif*) or h-index* or bibliometric* or altmetric* or Science of Implementation* or bibliography of medicine)

11 (MM "Health Services Research")

12 (MM "Translational Medical Research")

13 (MM "Research, Interdisciplinary")

14 9 or 10 or 11 or 12 or 13

15 8 and 14

**Limiters** - Published Date: 19700101-20231131; English Language
